# Synergistic Co-Inoculation of *Bacillus velezensis* and *Pseudomonas helmanticensis* Enhances Corn Straw Degradation via Microbial Community Restructuring and Saprotroph Dominance

**DOI:** 10.3390/microorganisms13112612

**Published:** 2025-11-17

**Authors:** Shihao Wang, Chang Su, Siqi Yang, Shuai Wang, Xingtong Jiang, Hongli He, Jianfeng Liu, Yunqing Cheng

**Affiliations:** Jilin Provincial Key Laboratory of Plant Resource Science and Green Production, Jilin Normal University, Siping 136000, China; 15844095925@163.com (S.W.); 15584585118@163.com (C.S.); 15943050987@163.com (S.Y.); 18843403380@163.com (S.W.); lion1259971664@163.com (X.J.); honglihe2002@126.com (H.H.);

**Keywords:** microbial consortia, straw degradation, soil microbial community, saline-alkali soils

## Abstract

This study evaluated the potential of individual and co-inoculation with *Bacillus velezensis* (Bv) and *Pseudomonas helmanticensis* (Ph) as microbial decomposers for corn straw. The co-inoculation (BP) treatment demonstrated the highest total mass loss for cellulose, hemicellulose, and total straw, significantly outperforming the single-strain treatments (Bv and Ph) and the non-inoculated control (CK). All inoculated treatments consistently enhanced degradation over time and lowered pH compared to CK. High-throughput sequencing revealed that inoculation dramatically reshaped the soil microbial community. All treatments reduced microbial ASVs and bacterial alpha diversity (ACE, Chao1, Shannon), with the most pronounced effect observed for Bv. Beta diversity analysis showed distinct, treatment-specific clustering. Critically, FUNGuild analysis indicated a significant functional shift, with all inoculants increasing saprotroph abundance and decreasing pathotrophs. The BP consortium exhibited a synergistic effect, driving saprotroph dominance to >96%. These results demonstrate that the synergistic co-inoculation enhances straw decomposition by restructuring the microbial community towards functional dominance of saprotrophs.

## 1. Introduction

As the world’s largest agricultural producer, China generates over 6.10 × 10^10^ tons of crop residues annually, with maize stover predominating in the northeastern Corn Belt [1]. This aligns with global cereal systems generating approximately 250 million metric tons of straw per annum [2]. Although straw incorporation enhances soil organic carbon (SOC) sequestration, improves aggregate stability, and promotes ecological intensification [3], its implementation in Northeast China faces critical biophysical limitations. Subfreezing winter temperatures (<−20 °C) severely inhibit enzymatic decomposition kinetics, resulting in partially degraded residues that serve as overwintering reservoirs for phytopathogens—a phenomenon associated with 20–40% increased disease incidence in subsequent growing seasons [4]. Conventional open-field burning exacerbates atmospheric PM_2.5_ pollution and respiratory morbidity [5], while the recalcitrant lignocellulosic matrix (lignin: 15–25%; cellulose: 35–50%) intrinsically limits natural decomposition efficiency [6].

To address these challenges, microbial inoculation has emerged as a promising strategy to accelerate straw decomposition under adverse conditions, primarily through well-documented mechanisms [7,8]. Feasibility is demonstrated by studies where inoculants containing cellulolytic and ligninolytic microorganisms significantly enhanced straw degradation rates even in cool climates [7]. The main mechanisms involve the secretion of a consortium of extracellular enzymes, such as lignin peroxidase (LiP), manganese peroxidase (MnP), and cellulases, which collaboratively break down the complex lignocellulosic structure [8,9]. Furthermore, introduced microbes can restructure the native soil microbial community, favoring saprotrophic taxa that dominate decomposition processes, thereby creating a self-sustaining degradative environment [10]. Notably, synergistic consortia often outperform single-strain inoculants by providing complementary enzymatic activities and ecological niches [11]. However, the efficacy of specific bacterial partnerships, particularly between genera like *Bacillus* and *Pseudomonas*, in restructuring the soil microbiome to favor saprotrophs for enhanced corn straw decomposition remains underexplored, especially in the context of Northeast China’s unique agro-climatic conditions.

While numerous microbial consortia have been developed, showing capabilities to degrade a significant portion of straw within several months [12,13], the underlying mechanisms, particularly how they reshape the soil microbial community to achieve a functional dominance of decomposers, are not fully understood. For instance, the specific interactions within a bacterial partnership (e.g., *Bacillus* and *Pseudomonas*) and how they collectively steer the native microbial community toward a saprotroph-dominated state to enhance decomposition remain critical knowledge gaps [14,15]. Elucidating this community-level mechanism is key to developing more effective and predictable microbial agents.

Recent studies have advanced the development of functional microbial consortia for straw degradation. For instance, a recent study demonstrated the efficacy of a cellulolytic consortium in accelerating decomposition [9], while bacterial-fungal synergies have also been highlighted as crucial for lignocellulose breakdown [10]. A critical frontier lies in understanding how a designed consortium can simultaneously achieve multi-dimensional agro-ecosystem benefits—specifically, coupling efficient straw degradation with pathogen suppression and soil amelioration—through a defined mechanism of microbial community restructuring. The potential of a minimal, well-defined bacterial consortium (e.g., *Bacillus* and *Pseudomonas*) to drive a functional shift in the soil microbiome towards near-complete saprotroph dominance, thereby concurrently addressing waste management and plant health, remains a significant knowledge gap. Our study is designed to fill this gap.

Based on the complementary enzymatic potentials of *Bacillus velezensis* (Bv, cellulolytic) and *Pseudomonas helmanticensis* (Ph, ligninolytic), we hypothesized that their co-inoculation would act synergistically to enhance corn straw degradation beyond the efficacy of either strain alone. We further hypothesized that this enhancement would be driven not only by their direct enzymatic actions but also through a fundamental restructuring of the indigenous soil microbial community, leading to a functional dominance of saprotrophic taxa that specialize in decomposition. To test these hypotheses, the objectives of this study were: (1) To quantitatively compare the efficiency of lignocellulose degradation (cellulose, hemicellulose, and total straw mass loss) among individual inoculations (Bv or Ph) and their co-inoculation (BP). (2) To assess the impact of these inoculations on the structure and diversity of the straw-associated soil bacterial and fungal communities. (3) To determine the shifts in the functional guilds of the fungal community, specifically testing whether inoculations increase the relative abundance of saprotrophs. (4) To correlate the changes in microbial community structure and function with the observed straw degradation kinetics, thereby elucidating the potential mechanism behind the synergistic effect. Through controlled microcosm experiments, this work aims to provide a mechanistic understanding of how bacterial consortia enhance straw decomposition by manipulating the soil microbiome. Therefore, the primary objective of this study was to assess and compare the efficacy of different inoculation treatments (Bv, Ph, and BP) at key stages of straw degradation (15, 30, and 45 days), with a focus on their impacts on lignocellulose decomposition, soil pH, and the structure and function of the microbial community. Our findings will contribute to developing effective microbial agents for sustainable agricultural waste management.

## 2. Materials and Methods

### 2.1. Lignocellulose-Degrading Microorganisms

Given the recalcitrant nature of agricultural straw containing 30–40% lignin [16], two microbial strains with lignocellulolytic potential were selected from the culture collection of Jilin Normal University’s Microbiology Research Laboratory (43°9′ N, 124°20′ E): *Bacillus velezensis* (CGMCC 20507), *Pseudomonas helmanticensis* (isolated from *Scolytus seulensis* gut microbiota).

### 2.2. Determination and Analysis of Degradation Indicators

**Figure 1:**
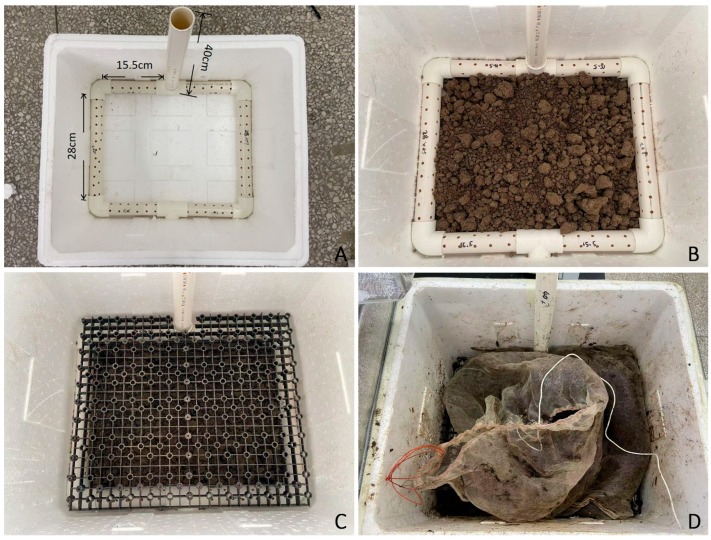
Schematic of the custom aerobic fermentation system used in this study. (**A**) Ventilation pipes at the system base. (**B**) Bottom layer covered with soil. (**C**) Spring spacer plate installed above the basal soil layer. (**D**) Nylon bags containing straw, microbial inoculants, and soil mixture arranged atop the spacer plate.

The aerobic fermentation system was constructed using an expanded polystyrene cultivation chamber (410 × 360 × 290 mm^3^) integrated with PVC piping components. Specifically, 1.58 m of PVC ventilation pipes (25 mm inner diameter) was precision-cut into four 15.5 cm segments, two 28 cm segments, and one 40 cm segment using an electric rotary saw. Thermal drilling was employed to create ventilation ports matching the outer diameter of four 90° elbow joints and two T-connectors, with the final assembly shown in Figure 1A. The chamber base was layered with 1.8 kg of non-sterile agricultural soil (particle size < 2 mm) compacted to form a 10 cm uniform layer (Figure 1B,C), and overlaid with a spring spacer plate maintaining 5 cm clearance from the soil surface.

The experiment comprised four treatments: (CK) 50 mL of sterile phosphate-buffered saline (PBS), (Bv) 50 mL of *B. velezensis* suspension (OD600 = 1.0) in PBS, (Ph) 50 mL of *P. helmanticensis* suspension in PBS, and (BP) 50 mL of a 1:1 mixture of both bacterial suspensions in PBS, using sterilized substrates containing 2 kg pulverized straw (<5 mm particles) and 500 g humic soil (45 ± 3% organic content) autoclaved at 121 °C for 30 min; inoculation protocols included: (CK) 50 mL sterile distilled water, (Bv) 50 mL *B. velezensis* suspension (OD_600_ = 1.0) prepared by centrifuging 600 mL culture (8000× *g*, 10 min) with pellet resuspended in phosphate-buffered saline, (Ph) identical protocol to Bv using *P. helmanticensis*, and (BP) combined 300 mL *B. velezensis* and 300 mL *P. helmanticensis* suspensions processed equivalently, with all inoculants pre-cultured in LB broth (30 °C, 180 rpm, 12 h); sampling at 15, 30, and 45 days post-inoculation generated 12 experimental groups (Bv-15 to BP-45) with three biological replicates per group (*n* = 36 samples). The substrate was sterilized to primarily investigate the degradative capability of the inoculated strains without the confounding competition from the native soil microbiome.

Substrate mixtures (straw: soil = 4:1 *w*/*w*) were moisture-adjusted to 35 ± 5% with sterile distilled water, then packed into 100-μm nylon mesh bags (20 × 30 cm) containing 500 g of material each (Figure 1D). These were incubated under ambient conditions (25 ± 3 °C, 55 ± 10% RH) for 15, 30, and 45 days. At each interval, samples were processed as follows: 10 g of homogenized material was freeze-dried with a Christ Alpha 1-4 LDplus freeze dryer (Martin Christ Gefriertrocknungsanlagen GmbH, Osterode am Harz, Germany), ball-milled (Retsch GmbH, Haan, Germany), and analyzed cellulose, hemicellulose, and lignin fractions of the samples were quantified using a sequential gravimetric method based on fiber fractionation [17]. Soil pH was determined by mixing 1 g of fresh sample with 5 mL of deionized water (vortexed for 30 s and settled for 12 h) using a calibrated pH meter (Mettler Toledo SevenExcellence; Mettler-Toledo GmbH, Greifensee, Switzerland). After the degradation period, the entire content of each bag was transferred to a sieve and thoroughly rinsed with tap water to remove all soil particles. The retrieved corn stover residues were then oven-dried at 65 °C to constant weight to determine the dry mass (W_1_). The total mass loss of corn stover was calculated using the formula: Corn stover total mass loss (%) = [(W_0_ − W_1_)/W_0_] × 100%, where W_0_ denotes the initial dry mass (g) of the corn stover before degradation. In this study, the soil used in the bottom layer of the fermentation system shares the same source as the soil incorporated into the mixture (containing soil, straw, and microbial inoculants). The physiochemical properties are listed as follows: pH = 6.67; Organic matter 12.4 g/kg; Total N 89 g/kg; Total P 20.4 g/kg; NH_4_OAc-K 197 mg/kg.

### 2.3. Soil DNA Extraction and Sequencing

From each experimental replicate, 5 g soil subsamples were aseptically collected from the substrate-nylon bag interface within the aerobic fermentation system. The 36 specimens were cryopreserved in liquid nitrogen (1 h) and maintained on dry ice during transport to Beijing Biomarker Technologies Co., Ltd. (Beijing, China) for analysis. Total genomic DNA was extracted using the E.Z.N.A.^®^ Soil DNA Kit (OMEGA Bio-Tek, Norcross, GA, USA) with enhanced mechanical lysis (0.1 mm zirconia beads, 6 m/s, 45 s). Domain-specific amplification targeted bacterial communities using 16S rRNA V3-V4 primers 338F (5′-ACTCCTACGGGAGGCAGCA-3′)/806R (5′-GGACTACHVGGGTWTCTAAT-3′) and fungal communities via ITS1 primers ITS1F (5′-CTTGGTCATTTAGAGGAAGTAA-3′)/ITS2R (5′-GCTGCGTTCTTCATCGATGC-3′) under standardized thermal cycling conditions (95 °C/3 min; 30 cycles: 95 °C/30 s, 55 °C/30 s, 72 °C/45 s; final extension 72 °C/10 min). Purified amplicons (AxyPrep Mag PCR Clean-up System, Axygen, Union City, CA, USA) were quantified using a Qubit 4.0 Fluorometer (dsDNA HS Assay Kit, Thermo Fisher Scientific, Waltham, MA, USA), normalized, and constructed into sequencing libraries. These libraries were sequenced on an Illumina NovaSeq 6000 platform (San Diego, CA, USA) (2 × 250 bp paired-end) with 10% PhiX spike-in controls. Bioinformatics processing involved quality trimming with Trimmomatic v0.33 [18] (4 bp sliding window, Q20 threshold) to remove low-quality bases. Primer removal and elimination of poly-G tails (a known artifact of NovaSeq chemistry) were performed simultaneously using Cutadapt v1.9.1 (max 10% mismatch; poly-G trimming enabled). Subsequent steps included denoising (truncLen F245/R220, maxEE = 2), paired-end merging (≥20 bp overlap), and chimera removal via DADA2 [19] implemented in QIIME2 v2020.6.0 [20], ultimately generating high-confidence amplicon sequence variants (ASVs).

### 2.4. Sequencing Data Analysis

ASVs, representing biologically meaningful sequences, enable discrimination at single-nucleotide resolution for precise species- or strain-level taxonomic identification. ASVs were generated using DADA2 [19] within QIIME2 v2020.6.0 [20] for sequence denoising. Taxonomic annotation of ASVs sequences was performed using a naive Bayes classifier trained on the SILVA reference database (release 138.1, QIIME compatible version), achieving species-level classification. This facilitated comprehensive community composition analysis across all taxonomic ranks (phylum to species). Species abundance matrices at each taxonomic level were compiled in QIIME2.

Compositional data were visualized using GraphPad Prism v10.1.2 (accessed: 14 April 2025) and ChiPlot (https://www.chiplot.online, accessed on 19 April 2025). Alpha diversity (within-sample richness/diversity) was assessed using the Chao1 [21], ACE [22], Shannon [23], and Simpson [24] indices. Beta diversity, representing between-sample compositional dissimilarity, was calculated in QIIME2 using four distance metrics: binary Jaccard, Bray–Curtis, weighted UniFrac, and unweighted UniFrac. Pairwise dissimilarity between samples was computed using the binary Jaccard algorithm [25] and visualized as heatmaps in ChiPlot, with color gradients indicating dissimilarity levels.

Fungal functional guilds were classified using FUNGuild (http://www.funguild.org; accessed on 1 May 2025), categorizing sequences into ecological functional groups (pathotrophs, symbiotrophs, saprotrophs) based on trophic modes. Fungal functional phenotypes were predicted via BMKCloud (www.biocloud.net). The experiment included 36 samples divided into four groups (*n* = 9 per group): Group CK (Control): CK15, CK30, CK45 (3 biological replicates each); Group Bv: Bv15, Bv30, Bv45 (3 biological replicates each); Group Ph: Ph15, Ph30, Ph45 (3 biological replicates each); Group BP: BP15, BP30, BP45 (3 biological replicates each).

### 2.5. Statistical Analysis

Statistical analyses were performed using SPSS 26.0 (IBM Corp., Armonk, NY, USA). Continuous variables were compared across experimental groups using one-way analysis of variance (ANOVA). Where ANOVA indicated significant differences (*p* < 0.05), Fisher’s Least Significant Difference (LSD) post hoc tests were applied for pairwise comparisons. Statistical significance was defined at *α* = 0.05.

Statistical analyses were performed using SPSS 26.0 (IBM Corp., USA). Given that the core aim of this study was to compare the effects of different treatments at each specific time point rather than to model the temporal progression, continuous variables for each sampling time (day 15, 30, and 45) were compared across the four experimental groups using one-way analysis of variance (ANOVA). Where ANOVA indicated significant differences (*p* < 0.05), Fisher’s Least Significant Difference (LSD) post hoc tests were applied for pairwise comparisons. Statistical significance was defined at α = 0.05. It is noteworthy that while repeated-measures ANOVA or a split-plot design represents a valid alternative for analyzing temporal data, the approach employed here is statistically appropriate and directly addresses the primary research question of treatment efficacy at discrete time points.

## 3. Results

### 3.1. Effects of Inoculants on Corn Straw Degradation and pH Dynamics

**Figure 2:**
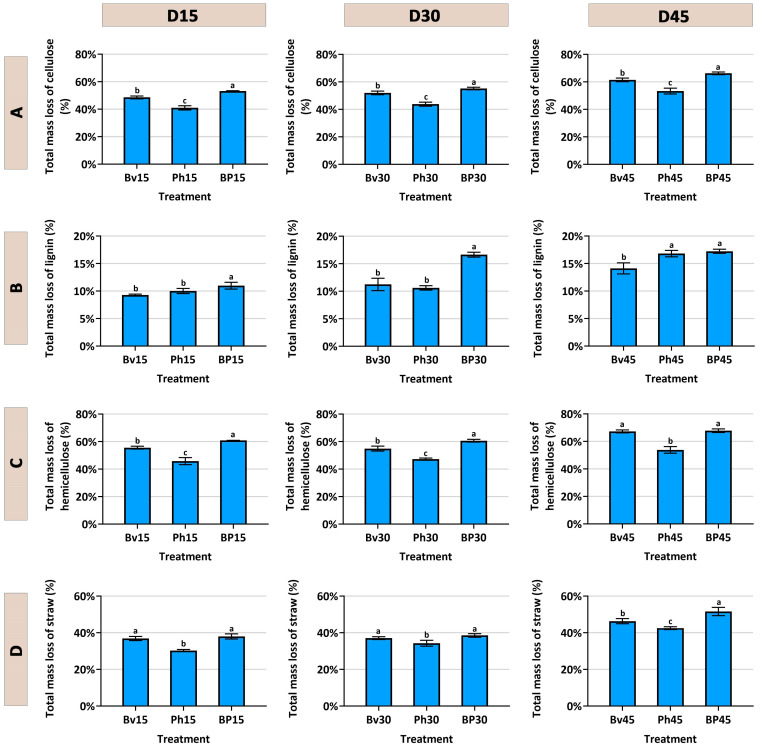
Temporal changes in straw degradation. Dynamics of (**A**) cellulose, (**B**) lignin, (**C**) hemicellulose, and (**D**) total straw mass loss over 45 days. Treatments: *Bacillus velezensis* (Bv), *Pseudomonas helmanticensis* (Ph), and co-inoculation (BP). Different lowercase letters indicate statistically significant differences between treatments at a given time point (*p* ≤ 0.05).

**Figure 3:**
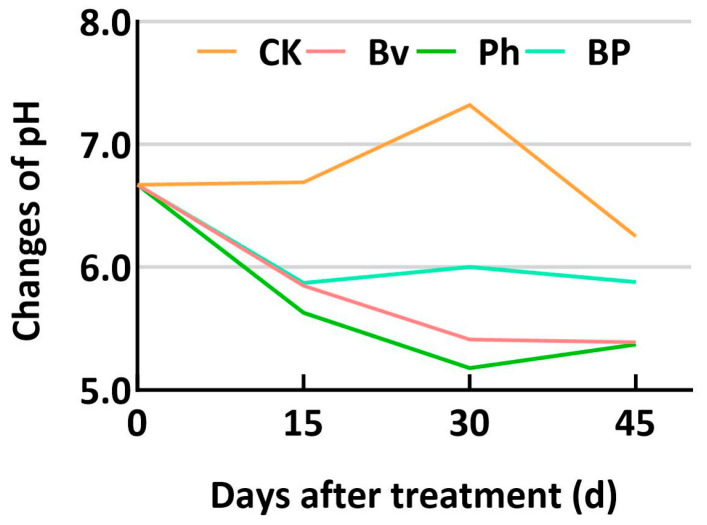
pH dynamics during fermentation. Abbreviations: CK, non-inoculated control; Bv, *Bacillus velezensis* monoinoculation; Ph, *Pseudomonas helmanticensis* monoinoculation; BP, *B. velezensis* + *P. helmanticensis* co-inoculation. Suffixes (15, 30, 45) indicate days post-inoculation (d).

The degradation kinetics of corn straw components were markedly influenced by the inoculation treatments over the 45-day incubation period (Figure 2). As intended by the substrate sterilization, the non-inoculated control (CK) exhibited minimal degradation across all components, confirming the effective suppression of the native microbiome and highlighting the essential role of the inoculated strains in the decomposition process. Consistent with the study’s theme of synergy, the co-inoculation treatment (BP) consistently demonstrated the most efficient and significant (*p* ≤ 0.05) degradation of cellulose (Figure 2A), hemicellulose (Figure 2C), lignin (Figure 2B), and consequently, the total corn straw mass (Figure 2D) at all time points compared to the CK and monoinoculation treatments. Among the single-strain inoculants, *Bacillus velezensis* (Bv) enhanced the degradation of all components more effectively than *Pseudomonas helmanticensis* (Ph), which resulted in the lowest degradation efficacy among the inoculants. During fermentation, the pH value in the control (CK) showed an initial increase followed by a decrease. Compared to CK, the pH values in the Bv, Ph, and BP treatments were consistently lower at the same fermentation days, with differences ranging from 0.32 to 2.14 (Figure 3). Consequently, Bv, Ph, and BP treatments all effectively reduced the pH of the corn straw.

### 3.2. Raw Data Quality Control and OTU/ASV Analysis

A total of 36 samples were sequenced, generating 2,483,671 raw reads (deposited in NCBI SRA under accession PRJNA1257853). After quality control and paired-end read merging, 2,221,015 clean reads were retained. Subsequent denoising, paired-end sequence merging, and chimera removal resulted in 1,697,057 high-quality reads, with an average of 47,140 non-chimeric reads per soil sample (Table S1). The total ASVs count reached 67,018 with 1,679,930 ASV reads (Table S2), corresponding to per-sample averages of 1862 ASVs and 46,665 ASV reads. Read distribution across taxonomic levels is detailed in Table S3.

### 3.3. Effects of Different Microbial Inoculants on ASVs Number of Soil Samples

**Figure 4:**
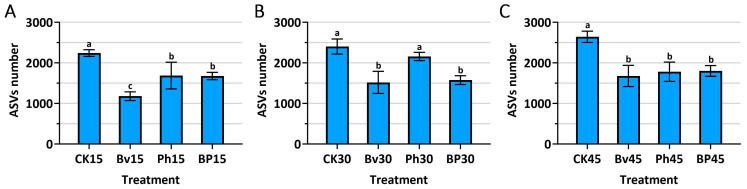
Soil microbial richness (Amplicon Sequence Variants, ASVs) in response to inoculation. (**A**) 15th day; (**B**) 30th day; (**C**) 45th day post-inoculation. Different letters above bars indicate significant differences between treatments at each time point (*p* ≤ 0.05). Abbreviations: CK, non-inoculated control; Bv, *Bacillus velezensis* monoinoculation; Ph, *Pseudomonas helmanticensis* monoinoculation; BP, *B. velezensis* + *P. helmanticensis* co-inoculation. Suffixes (15, 30, 45) indicate days post-inoculation (d).

Microbial diversity sequencing was performed on 36 soil samples, identifying 67,018 ASV sequences (OTU/ASV) as shown in Figure 4. The inoculation treatments exerted a profound and sustained impact on the soil microbial community richness, as quantified by the number of Amplicon Sequence Variants (ASVs) (Figure 4A–C). Contrary to the typical effect of nutrient addition, all bacterial inoculations—Bv, Ph, and BP—resulted in a significant reduction (*p* ≤ 0.05) in ASV richness compared to the non-inoculated control (CK) at every sampling time point (15, 30, and 45 days). This suggests a strong inhibitory effect of the introduced strains on the background microbial diversity that re-established post-sterilization. Notably, the *B. velezensis* monoinoculation (Bv) consistently induced the most severe reduction in microbial richness, displaying the lowest ASV counts across the entire experiment. The co-inoculation treatment (BP), while also significantly reducing richness compared to CK, consistently maintained a higher number of ASVs than the Bv treatment, indicating a moderated effect on the microbial community. This intermediate level of richness in the BP treatment, which corresponded with the most effective straw degradation (as shown in Figure 2), may reflect a more balanced and functionally optimal microbial environment conducive to decomposition.

### 3.4. Effects of Microbial Inoculants on the Relative Abundance of Soil Bacteria

ASV sequences from 36 soil samples represented 49 phyla, 126 classes, and 2121 genera (Table S4).

**Figure 5:**
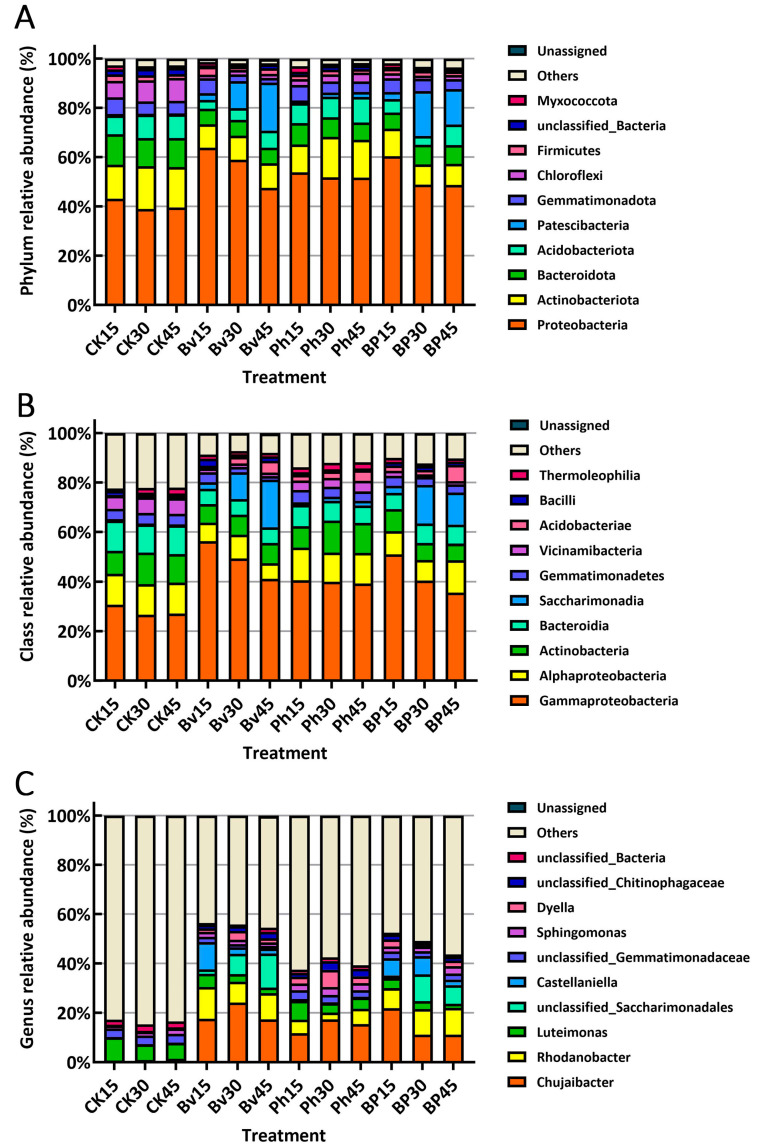
Microbial community composition at (**A**) phylum, (**B**) class, and (**C**) genus levels. Only the top 10 most abundant taxa are shown for each taxonomic rank. Abbreviations: CK, non-inoculated control; Bv, *Bacillus velezensis* monoinoculation; Ph, *Pseudomonas helmanticensis* monoinoculation; BP, *B. velezensis* + *P. helmanticensis* co-inoculation. Suffixes (15, 30, 45) indicate days post-inoculation (d).

At the phylum level, *Proteobacteria* exhibited the highest relative abundance (mean 50.60%), followed by *Actinobacteriota* (12.30%) and *Bacteroidota* (8.37%) (Figure 5A). Compared to the control (CK), the relative abundance of *Proteobacteria* was significantly lower in CK (40.57%) than in the Bv (56.77%), Ph (52.42%), and BP (52.63%) treatments. Conversely, *Actinobacteriota* relative abundance was significantly higher in CK (15.80%) than in the Bv (9.75%), Ph (14.35%), and BP (9.29%) treatments. Similarly, *Bacteroidota* relative abundance was significantly higher in CK (11.85%) than in the Bv (6.31%), Ph (7.88%), and BP (7.44%) treatments (Figure 5A). Collectively, the Bv, Ph, and BP treatments significantly increased the relative abundance of *Proteobacteria* in maize straw while reducing the relative abundances of *Actinobacteriota* and *Bacteroidota* to varying degrees.

At the class level, *Gammaproteobacteria* displayed the highest relative abundance (mean 39.84%), followed by *Alphaproteobacteria* (10.75%) and *Actinobacteria* (9.43%) (Figure 5B). Compared to the control (CK), *Gammaproteobacteria* relative abundance was markedly lower in CK (28.17%) than in the Bv (48.92%), Ph (39.90%), and BP (42.37%) treatments. *Alphaproteobacteria* relative abundance in CK (12.39%) resembled that in the Ph treatment (12.52%) but exceeded levels in Bv (7.85%) and BP (10.24%) treatments, while *Actinobacteria* relative abundance in CK (11.26%) was comparable to Ph (11.16%) but significantly higher than in Bv (7.85%) and BP (7.48%) treatments (Figure 5B). Collectively, Bv, Ph, and BP treatments significantly increased *Gammaproteobacteria* relative abundance in maize straw, whereas Bv and BP treatments reduced *Alphaproteobacteria* and *Actinobacteria* abundances.

At the genus level, *Chujaibacter* exhibited the highest relative abundance (mean 12.47%), followed by *Rhodanobacter* (6.40%) and *Luteimonas* (4.80%) (Figure 5C). Compared to the control (CK), *Chujaibacter* abundance was substantially lower in CK (0.65%) than in Bv (19.72%), Ph (14.80%), and BP (14.71%) treatments. Likewise, *Rhodanobacter* abundance in CK (0.31%) was markedly lower than in Bv (10.62%), Ph (4.87%), and BP (9.79%) treatments. In contrast, *Luteimonas* abundance was significantly higher in CK (7.39%) than across all inoculation treatments (Bv: 3.57%; Ph: 5.29%; BP: 2.97%) (Figure 5C). Collectively, Bv, Ph, and BP treatments significantly increased *Chujaibacter* and *Rhodanobacter* abundances while reducing *Luteimonas* abundance variably in maize straw.

**Figure 6:**
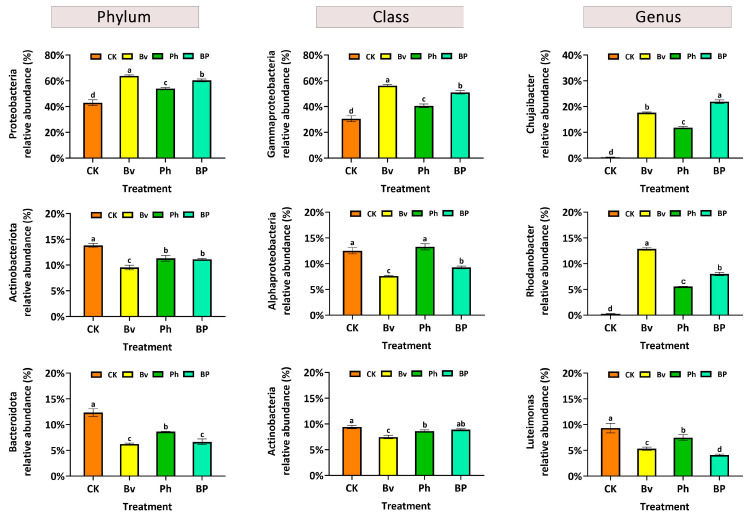
Relative abundance of the top three bacterial taxa at the phylum, class, and genus levels on day 15. Different letters indicate significant differences (*p* ≤ 0.05) among treatments across all time points. Abbreviations: CK, non-inoculated control; Bv, *Bacillus velezensis* monoinoculation; Ph, *Pseudomonas helmanticensis* monoinoculation; BP, *B. velezensis* + *P. helmanticensis* co-inoculation.

Analysis of the most abundant microbial taxa revealed significant shifts driven by both inoculation treatment and decomposition time (Figure 6, Figures S1 and S2). Regarding the treatment effect, inoculation, particularly the co-inoculation (BP), markedly increased the relative abundance of the predominant taxa, such as *Chujaibacter* and *Rhodanobacter*, which were consistently present at significantly higher levels in the BP, Bv, and Ph treatments compared to the control (CK) at days 30 and 45. Regarding the temporal effect, the relative abundance of these dominant taxa showed a substantial increase over time. Their abundance was generally low and similar across all treatments at the initial stage (day 15), but dramatically increased and diverged significantly among treatments by days 30 and 45, indicating a strong time-dependent enrichment driven by the inoculation.

### 3.5. Effects of Microbial Inoculants on the Alpha and Beta Diversity of Soil Bacteria

**Figure 7:**
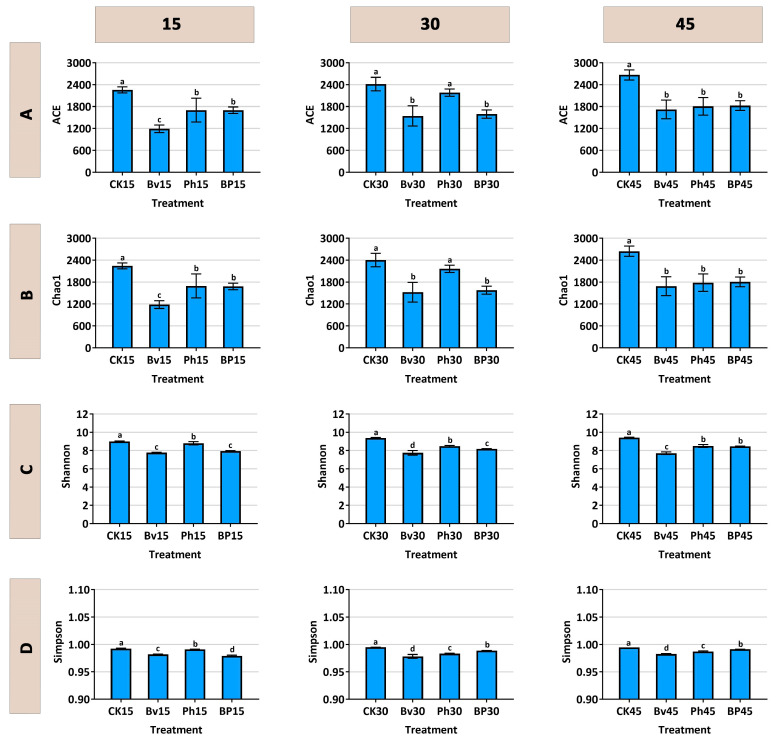
Soil bacterial alpha-diversity indices under different inoculation treatments. (**A**) ACE index, (**B**) Chao1 index, (**C**) Simpson index, (**D**) Shannon index. Soils were sampled at 15, 30, and 45 days post-inoculation. Treatments: control (CK), *B. velezensis* (Bv), *P. helmanticensis* (Ph), and co-inoculation (BP). Different lowercase letters above bars indicate statistically significant differences among treatments at each time point (*p* ≤ 0.05).

Soil bacterial alpha diversity was significantly influenced by the inoculation treatments (Figure 7A–D). Higher values of these indices generally indicate greater microbial diversity and richness. Throughout the experimental period, the non-inoculated control (CK) consistently exhibited the highest values in richness (ACE and Chao1) and Shannon diversity indices, all of which were significantly greater (*p* < 0.05) than those in any of the inoculated treatments. Among the inoculants, a clear gradient was observed: the *B. velezensis* monoinoculation (Bv) resulted in the most substantial reduction in alpha diversity, followed by the co-inoculation (BP), while the *P. helmanticensis* (Ph) treatment showed the least suppressive effect, yielding the order CK > Ph > BP > Bv.

Notably, this suppression of taxonomic diversity correlated with enhanced lignocellulose degradation (Figure 2D). The pronounced diversity reduction in the Bv treatment, which corresponded with high degradation efficiency, suggests that a simplified, saprotroph-dominated community may be more functionally specialized for straw decomposition. The co-inoculation (BP) achieved an optimal balance, maintaining strong degradation performance while moderating the impact on community diversity, highlighting the synergistic effect of combining these two strains.

**Figure 8:**
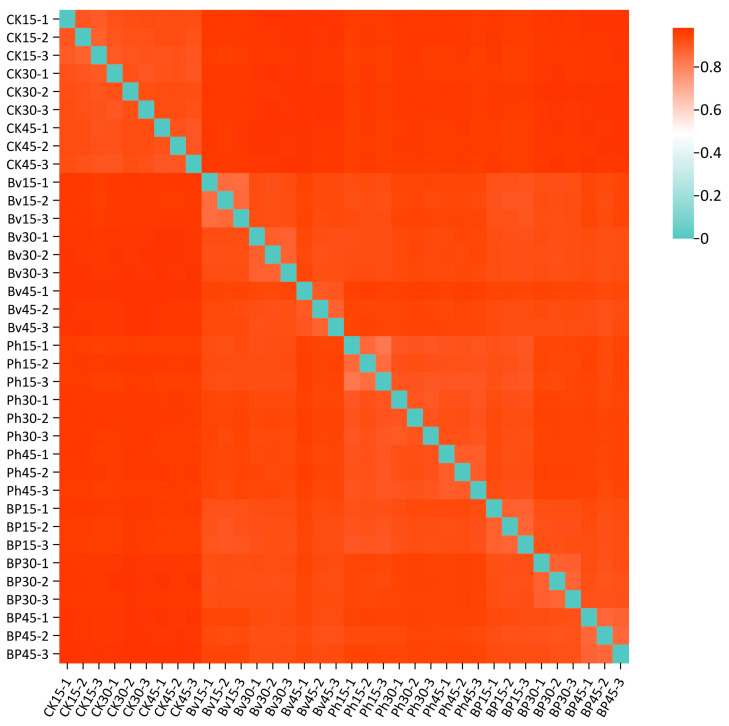
Heatmap of microbial community dissimilarity (Bray–Curtis distance). The color gradient from blue to red indicates increasing dissimilarity between samples. Treatments: control (CK), *B. velezensis* (Bv), *P. helmanticensis* (Ph), and co-inoculation (BP). Each sample label combines treatment and time point (e.g., Bv15_1: *B. velezensis*, 15 days, replicate 1).

Beta diversity analysis, visualized through inter-sample dissimilarity heatmaps (Figure 8), revealed distinct clustering patterns reflecting compositional heterogeneity across taxonomic levels. Samples exhibiting similar color profiles demonstrated higher community structure similarity. The heatmap showed tight clustering among the nine CK replicates, while Bv, Ph, and BP treatments formed separate clusters comprising 27 inoculated samples. This divergence indicates significant microbial community restructuring induced by the inoculations. Furthermore, high within-treatment homogeneity was observed across all inoculated groups (9 Bv replicates, 9 Ph replicates, 9 BP replicates), demonstrating convergent shifts in soil microbial assemblages following bacterial amendment.

**Figure 9:**
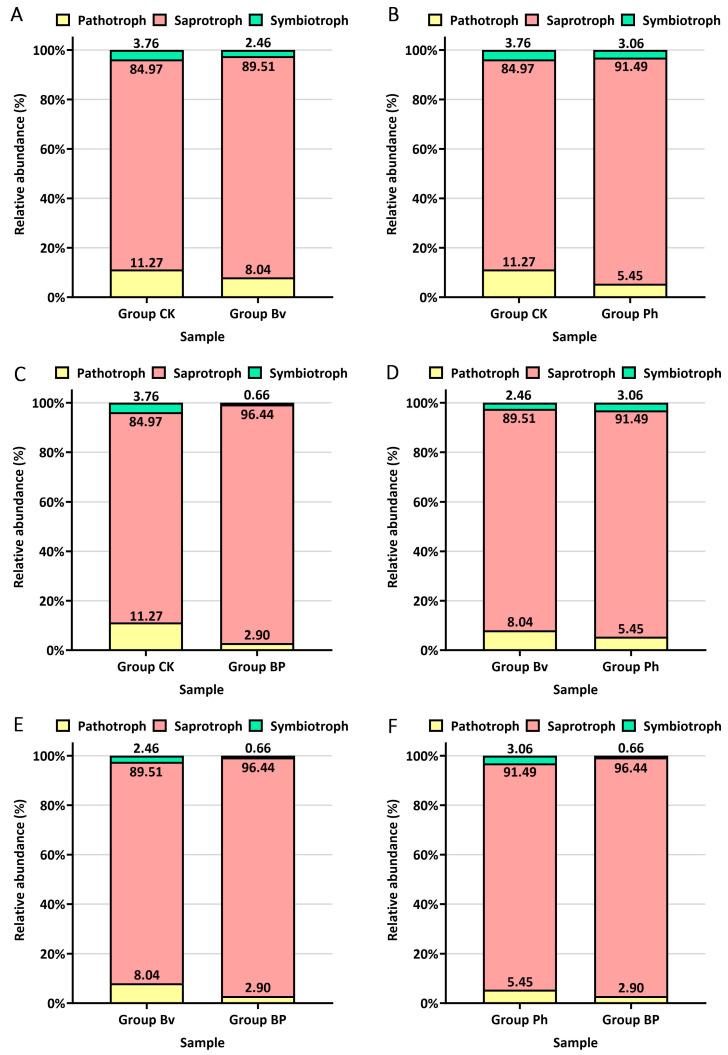
Functional guilds of fungal communities predicted by FUNGuild. Comparisons of the relative abundance of pathotrophs, symbiotrophs, and saprotrophs between different treatment groups: (**A**) CK vs. Bv, (**B**) CK vs. Ph, (**C**) CK vs. BP, (**D**) Bv vs. Ph, (**E**) Bv vs. BP, (**F**) Ph vs. BP.

FUNGuild (Fungal Functional Guild) is a tool for taxonomically parsing fungi based on ecological associations, enabling the classification of large sequence libraries into ecologically meaningful categories using a simple and consistent method. In this study, FUNGuild analysis and pairwise comparisons were performed on soil samples from four treatment groups: Group CK, Group Bv, Group Ph, and Group BP, with nine replicates per group. Across all soil samples, Saprotrophs exhibited the highest relative abundance (approximately 90%), followed by Pathotrophs (~7%), and Symbiotrophs (~3%) (Figure 9). Compared to the control (CK), the relative abundance of Saprotrophs was higher in the Bv (89.51%), Ph (91.49%), and BP (96.44%) treatments; the relative abundance of Pathotrophs was lower in the Bv (8.04%), Ph (5.45%), and BP (2.90%) treatments; the relative abundance of Symbiotrophs was lower in the Bv (2.46%), Ph (3.06%) and BP (0.66%) treatment than in CK (3.76%) (Figure 9A–C). Compared to the Bv treatment, the Ph treatment showed higher relative abundances of Pathotrophs and Symbiotrophs and a lower relative abundance of Saprotrophs (Figure 9D). Compared to the single-inoculation treatments (Bv and Ph), the BP treatment (mixed inoculation) showed a higher relative abundance of Saprotrophs (96.44% vs. Bv 89.51% and Ph 91.49%); a lower relative abundance of Pathotrophs (2.90% vs. Bv 8.04% and Ph 5.45%); and a lower relative abundance of Symbiotrophs (0.66% vs. Bv 2.46% and Ph 3.06%) (Figure 9E,F). Collectively, both Bv and Ph treatments increased Saprotroph abundance and decreased Pathotroph abundance relative to the control, though to varying degrees, while the mixed-inoculation BP treatment demonstrated an enhanced effect, with considerably higher Saprotroph abundance and lower Pathotroph abundance compared to the single-inoculation treatments.

## 4. Discussion

### 4.1. Enrichment of Functional Decomposer Guilds Drives Degradation Efficiency

Our microbiome analysis revealed a consistent restructuring toward a specialized decomposer community, aligning with the established concept of “niche modification” by keystone inoculants [26,27,28]. This phenomenon, wherein targeted introductions of specific strains steer the community towards a desired functional outcome, is a critical strategy in microbiome engineering [29,30]. The observed community simplification (Figure 4A–C) reflects a common principle in microbial ecology where functional specialization can override sheer diversity in achieving high process efficiency [31]. This principle is evident across diverse systems, from composting to the rhizosphere, where the structure and stability of microbial networks are shaped by key taxa.

The significant enrichment of acidogenic genera *Chujaibacter* and *Rhodanobacter* [32] (Figure 5C and Figure 6) provides a clear mechanistic link to straw breakdown. The proliferation of these genera, known for their robust metabolic capacities in organic matter decomposition [33,34], directly contributes to the acidification of the microenvironment (Figure 3). This acidification creates favorable conditions for the enzymatic hydrolysis of complex polymers like cellulose and hemicellulose [31,34], thereby enhancing the degradation efficiency observed across all inoculated treatments (Figure 2A–D). Conversely, the suppression of the acid-sensitive genus *Luteimonas* [35,36] (Figure 5C and Figure 6) further confirms that inoculation-induced niche modification selectively favors acid-tolerant decomposers. The parallel trends in acidification (Figure 3) and degradation efficiency (Figure 2) strongly suggest this causal pathway, though future metabolite-based studies are warranted for definitive validation.

Notably, the convergent community succession toward a predictable, saprotroph-dominated state (Figure 9), triggered even by single-strain inoculants, underscores the potency of initial perturbation. Shared mechanisms, such as acid secretion, can be sufficient to initiate significant ecological shifts [37,38]. Critically, the co-inoculation (BP) synergistically augmented *Chujaibacter* abundance (Figure 5C), suggesting complex ecological interactions including facilitation (where one strain modifies the environment to benefit the other) and potential syntrophy (metabolic cross-feeding that amplifies carbon flux through the decomposition network). This synergy exemplifies niche complementarity, wherein functionally distinct strains partition resources more efficiently than either could alone. The resulting saprotroph dominance exceeding 96% (Figure 9E,F) demonstrates how co-inoculation creates a more specialized decomposer community, optimizing the microbial network for straw decomposition—a finding consistent with established consortia studies [27,28].

### 4.2. Functional Specialization Outweighs Diversity in Driving Decomposition Efficiency

The reduction in bacterial alpha diversity across all inoculants (Figure 7A–D) is consistent with the concept of “specialization-driven diversity reduction” [39,40,41], commonly observed in high-resource, selective environments. This pattern does not indicate ecosystem impairment but rather reflects competitive exclusion of non-specialists and selection for decomposer specialists, leading to a highly efficient functional profile [42,43]. The pronounced diversity reduction in the Bv treatment, which nonetheless achieved high degradation efficiency (Figure 2), suggests that a simplified community structure may be more functionally specialized for straw decomposition.

Interestingly, the co-inoculation (BP) achieved an optimal balance, maintaining strong degradation performance (Figure 2D) while exhibiting a more moderate impact on community diversity compared to the Bv treatment (Figure 7). This intermediate level of taxonomic diversity, coupled with the highest functional specialization (Figure 9), highlights the synergistic effect of combining these two strains. The distinct clustering patterns in beta diversity analysis (Figure 8) further demonstrate the significant restructuring of microbial communities induced by the inoculations, with each treatment forming a unique ecological niche conducive to decomposition.

The overwhelming saprotroph dominance (>96% in BP treatment, Figure 9E,F) signifies a decisive metabolic shift toward decomposition specialization. This functional optimization, rather than taxonomic richness per se, appears to be the primary driver of enhanced degradation efficiency. These findings support the growing recognition that microbial community function is better predicted by functional traits than by diversity metrics alone, particularly in engineered systems designed for specific processes like lignocellulose decomposition. This optimal balance reflects the principle of niche complementarity, where complementary functional traits among co-inoculated strains enhance overall decomposition efficiency beyond what single strains can achieve, as observed in other engineered microbial systems [27,28].

## 5. Conclusions

This study conclusively validates our hypothesis that the *B. velezensis-P. helmanticensis* co-inoculant (BP) synergistically amplifies stover mineralization through tripartite mechanisms: (1) Enhanced stress resilience evidenced by BP’s superior total mass loss (cellulose: 66.21%; hemicellulose: 67.81%; total straw: 51.56%), exceeding single inoculants by 12–18%; (2) Ionic homeostasis facilitation via targeted pH reduction (ΔpH ≤ 2.14), generating an acidic microenvironment that activated acid-tolerant decomposers (*Rhodanobacter*) while suppressing salinity-sensitive competitors (*Luteimonas*); and (3) Functional pathogen suppression manifested through saprotroph dominance (>96%) and pathotroph depletion (4.14–5.54% reduction vs. CK). Critically, this synergy was microbiologically underpinned by competitive exclusion of non-functional taxa, reproducible community restructuring, and cross-kingdom functional guild convergence—culminating in BP’s unprecedented 96.44% saprotrophic dominance. Notably, these findings derive from controlled laboratory conditions where temperature, moisture, and substrate homogeneity were optimized; consequently, BP’s salinization mitigation capacity (Hypothesis ii) and field performance under variable soil matrices require validation in agricultural settings. These results highlight the potential of BP as a candidate for scalable straw valorization. However, prior to translational applications, future work must first establish the mechanistic linkage (e.g., the specific roles of acid-producing bacteria and their interactions with inoculants) underlying the community’s reproducible assembly and function. Following this, on-farm validation of degradation efficiency and salinity resilience, alongside integration of lignin-specialized Trichoderma to address current bottlenecks (9.27–17.22% lignin removal), will be prioritized. Thus, the initial hypotheses are fully confirmed: the co-inoculation acted synergistically to enhance degradation beyond the efficacy of single strains, and this enhancement was indeed driven by a fundamental restructuring of the microbial community towards functional saprotroph dominance, thereby elucidating the mechanism behind the observed synergy.

## Data Availability

The raw sequences of microbial diversity in soil can be found in the National Center for Biotechnology Information under the accession number PRJNA1257853. The original contributions presented in this study are included in the article/Supplementary Materials; further inquiries can be directed to the corresponding authors.

## Supplementary Materials

The following supporting information can be downloaded at: https://www.mdpi.com/article/10.3390/microorganisms13112612/s1, Table S1: Summary on raw data; Table S2: Feature number of each sample; Table S3: Statistics of reads count in each taxonomic level; Table S4: Statistics of taxonomic annotation; Figure S1: Relative abundance of the top three bacterial taxa at the phylum, class, and genus levels on day 30; Figure S2: Relative abundance of the top three bacterial taxa at the phylum, class, and genus levels on day 45.
