# Supplementary figures and images for: Synergistic Co-Inoculation of Bacillus velezensis and Pseudomonas helmanticensis Enhances Corn Straw Degradation via Microbial Community Restructuring and Saprotroph Dominance
Source: Microorganisms. 2025 Nov 17;13(11):2612. doi: 10.3390/microorganisms13112612 (PMC12654394; doi:10.3390/microorganisms13112612)

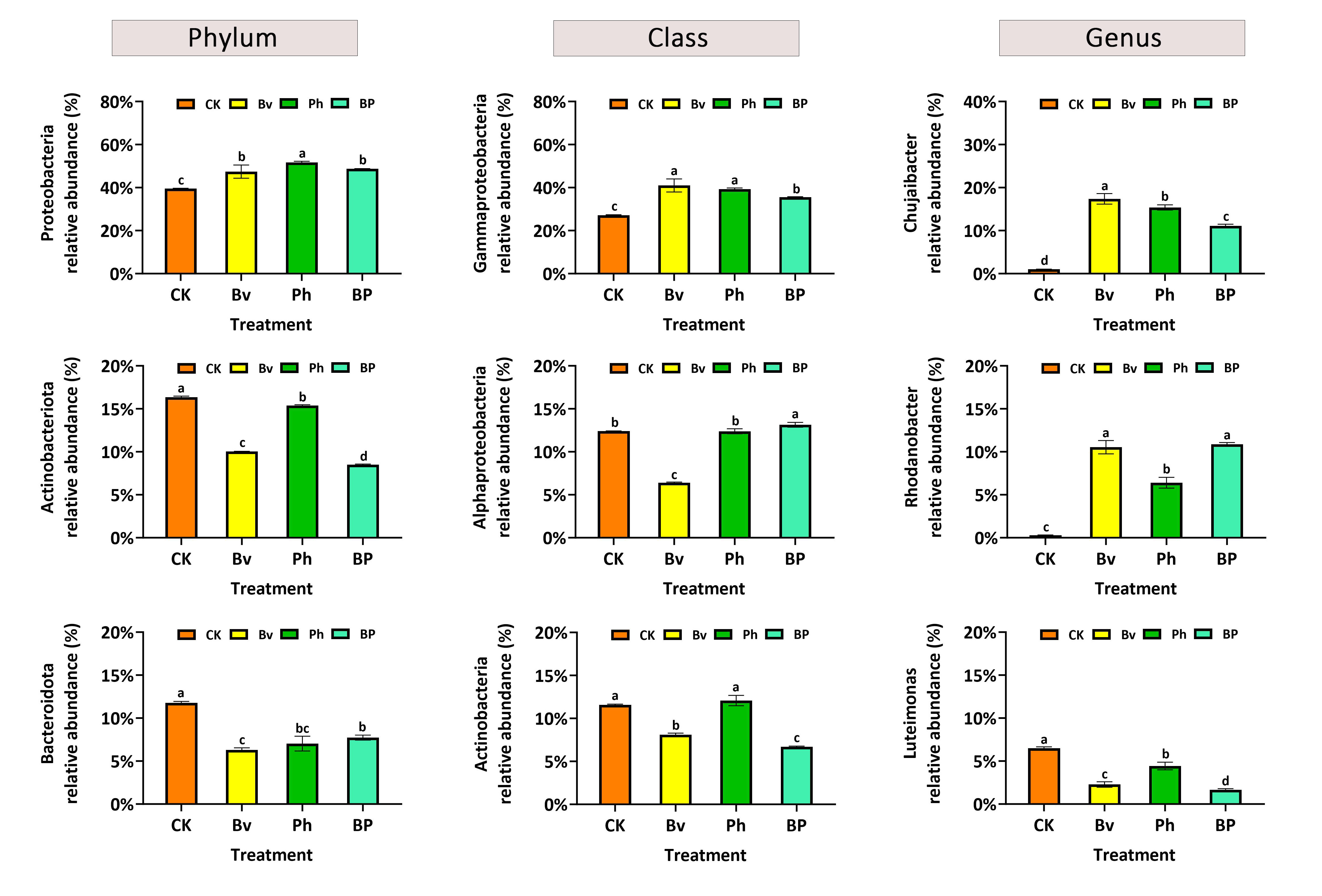

Supplement: Supplementary file 1 [file microorganisms-13-02612-s001.zip › microorganisms-3967029-supplementary/Figure S1 Relative abundance of the top three bacterial taxa at the phylum, class, and genus levels on day 30.jpg]
